# The avian community of the Karen Mogensen Reserve, a wealth of biodiversity within the poorly investigated and threatened environments of northwestern Costa Rica

**DOI:** 10.3897/zookeys.722.14606

**Published:** 2017-12-13

**Authors:** Matteo Dal Zotto, Giuseppe Romeo, Luis A. Mena Aguilar, Dario Sonetti, Aurora Pederzoli

**Affiliations:** 1 Department of Life Sciences, University of Modena and Reggio Emilia, via Campi, 213/d, I-41125 Modena, Italy; 2 Consortium for the Interuniversity Center of Marine Biology and Applied Ecology, viale N. Sauro, 4, I-57128 Livorno, Italy; 3 Associazione Foreste per Sempre, Via D’Avia Sud 65/a, Modena, Italy; 4 Asociación Ecológica Paquera, Lepanto y Cóbano, Jicaral, Nicoya, Costa Rica

**Keywords:** Aves, biogeography, birds, conservation biology, Mesoamerica, tropical ecology, tropical forests

## Abstract

Despite being characterized by some of the most threatened forest ecosystems of Mesoamerica, the Nicoya Peninsula is among the least known regions of neotropical Costa Rica in terms of its birdlife. Within this region, in the framework of an ongoing international cooperation program between Italy and Costa Rica, we had the opportunity to investigate the Karen Mogensen Reserve, a protected area distinguished by the presence of a variety of habitats, including tropical dry forest and moist forest. Species richness in the Reserve was relatively high compared with similar areas in northwestern Costa Rica. A series of surveys carried out over a 20-year period documented an avian community consisting of 207 species, of which 115 were breeding in the zone and another 14 were potentially breeding. We recorded five IUCN globally Vulnerable or Near-Threatened species, along with six species reported for the first time from the Nicoya Peninsula, each representing range extension of more than 100 km. Twenty-six species, mostly breeding in the area, are at their southernmost range borders, and are likely susceptible to global environmental alterations, such as the effects of climate change. Furthermore, our study revealed the presence of two species endemic to a restricted area of Central America and four subspecies endemic to Costa Rica, along with breeding populations of two species that are geographically isolated from the main ones. The present analysis led to the ecological characterization of the resident avian community, showing that 65% of the species are strictly associated with forested environments, and especially with the understory or middle tree level, hence more vulnerable to environmental change (climatic, anthropogenic, etc.) and susceptible to local extinction. These results underscore the importance of the Karen Mogensen Reserve for bird conservation within a vulnerable environmental context, and warrant the continuation of periodic bird surveys, taxonomic study of isolated populations or endemic taxa, and improvement of local conservation measures. The data collected will be an important tool for future studies aimed at evaluating the consequences of habitat fragmentation and to monitor the effects of climate change on the resident avifauna. We exhort the creation of programs that integrate bird monitoring, ecological research, conservation initiatives, and the involvement of the local communities, by promoting environmental education, capacity-building, and income generation. To this purpose, the Karen Mogensen Reserve may represent a convincing model and valuable example to apply in similar neotropical contexts.

## Introduction

Despite its relatively small size of approximately 51,000 km^2^, Costa Rica exhibits an extremely rich biodiversity, with more than 500,000 known species, corresponding to nearly 5% of the species estimated worldwide (INBio 2017). The avian checklist of the country accounts for 927 species: almost one tenth of those known worldwide (Gill and Donsker 2017, Lepage 2017). Over 600 species are resident, and the remaining are migratory (mainly from North America) or accidental (Stiles et al. 2003, Garrigues et al. 2015, Lepage 2017). Ten species are endemic to Costa Rica, approximately 80 show a limited distribution in Central America, and 26 species are threatened on a global scale (Lepage 2017), of which four are endangered (Stattersfield et al. 1998). The key of this richness derives firstly from tropical latitudes and the geographic position of Costa Rica, which is a natural bridge between North and South America, but also topography and its influence on climate plays an important role (Wege and Long 1995).

Movements of the tectonic plates over approximately the past ten million years led to the formation of four volcanic mountain ranges, aligned in a northwest-southeast orientation, which in some cases exceed 3,500 m in altitude. These ridges act as barriers to the passage of moist air, creating marked differences in rainfall and temperatures between the Pacific and Caribbean slopes of the country, and also between lowlands and highlands. This climate and habitat complexity is the basis for the presence of at least six main life zones (excluding the Cocos Island), each characterized by unique avian communities, namely: Northern Pacific and Southern Pacific lowlands and foothills, Middle elevations, Highlands, Caribbean lowlands and Caribbean foothills (Slud 1964, Reid et al. 2010, Garrigues and Dean 2014).

The extreme biological richness of Costa Rica, together with its favorable socio-political context, a national forest conservation program, and its regeneration policy, have drawn the attention of many scientists, especially during the last 50 years. Numerous surveys have contributed to the knowledge of the Costa Rican avifauna since the pioneering studies of Alexander von Frantzius and José Castulo Zeledón during the nineteenth century. At present, the bird life of the country is considered one of the best known in Central America (Garrigues and Dean 2014). Nevertheless, there are still various poorly explored locations, and detailed studies of birds and other metazoan groups are warranted in some parts of the country.

The Nicoya Peninsula, an important geological unit in the northwestern part of Costa Rica (Castillo-Muñoz 1977), is probably the least studied area of the country in terms of ornithological knowledge. Various authors have reported a lack of information for the greatest part of this region (Castro et al. 2001, Villareal Orias et al. 2003, Solano et al. 2004). The peninsula is situated in the northern Pacific lowlands and foothills life zone. Its main habitat is tropical dry forest, characterized by a rainy season followed by a strong desiccation of the vegetation from December to May, resulting from dry winds, constant sunshine, and lack of precipitation (Holdridge 1967, Gomez 1985, Herrera 1985, Mata and Echeverría 2004). Recently, it has been shown that local conditions affect the duration of the dry season at different sites of the region creating some local ecosystem variability (Sánchez-Murillo et al. 2013). Precipitation patterns influence the vegetation, which is dominated by deciduous taxa with evergreen species along rivers and streams (Hartshorn 1983). This seasonally dry climate and deriving habitats are typical of other Central American countries from Costa Rica up to Mexico. These environments are of high conservation interest because they are considered the most threatened forest ecosystems of Mesoamerica (Janzen 1988, Huettmann 2015). Birds living in such conditions normally show wide ranges, covering most of the Pacific slope of Central America. For this reason, many species inhabiting this life zone in Costa Rica tend to be at the southern limit of their range (Slud 1964). However, recent studies based on both morphological and molecular data have reported that at least some of these taxa, such as the Plain Wren (*Cantorchilus modestus*), are in fact species complexes (Saucier et al. 2015), thus warranting analyses of similar species complexes.

During the first half of the twentieth century, major parts of the Nicoya Peninsula were deforested and converted to cattle pasture. More generally, the northwestern Costa Rican dry forest was reduced to a series of small patches surrounded by large areas used for cattle pastures or crops (e.g., sugar cane) (Quesada and Stoner 2004). Although in its native state this land should be almost entirely forested, at present, the dry forest covers only 0.1% of its original extension (Janzen 1988). As a result, the terrestrial avifauna is confined to these isolated forest patches, with little prospect of reconnection (Barrantes et al. 2016). In fact, it has been shown that the populations of some species are restricted to the highest mountains or foothills of the Peninsula (Slud 1964).

One of these forest patches is the Karen Mogensen Reserve, an area located in the southeastern portion of the Peninsula. Since the foundation of the Karen Mogensen Reserve in 1996, some ornithological expeditions were conducted. The latest of these were in 2016 and 2017, after the opening of the research station “Italia-Costa Rica”. These expeditions were part of a project aimed at investigating the local avian community and the meteorological aspects of the area, in order to assess a possible correlation between biodiversity data and climate change. Here we provide the results of these surveys, along with an annotated checklist of the birds of the Karen Mogensen Reserve and the ecological characterization of the avian community, focusing on the phenological, biogeographic, and conservational aspects of the encountered species.

## Study area

**Figure 1.**
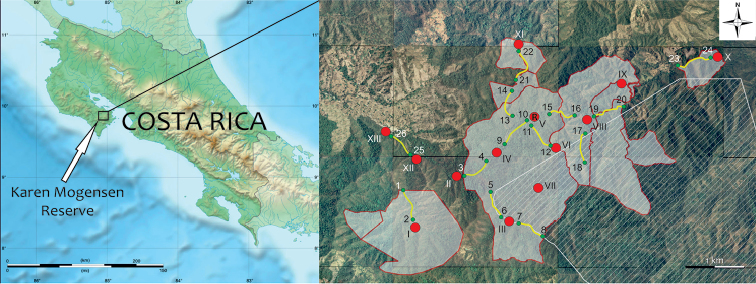
Map of Costa Rica and the Karen Mogensen Reserve. The areas in white, bordered by red lines, are parts of the Karen Mogensen Reserve added to the original nucleus over years. The area filled by a striped pattern represents the biological corridor of the Nicoya Peninsula and the additional protected forested land adjacent to the Karen Mogensen Reserve. “R” shows the position of the Research Station. Red dots are the fixed points, yellow lines are the transects used for the data collection during the surveys; green dots represent the transects endpoints. Numbers and Roman numerals refer to Table 1.

**Figure 2.**
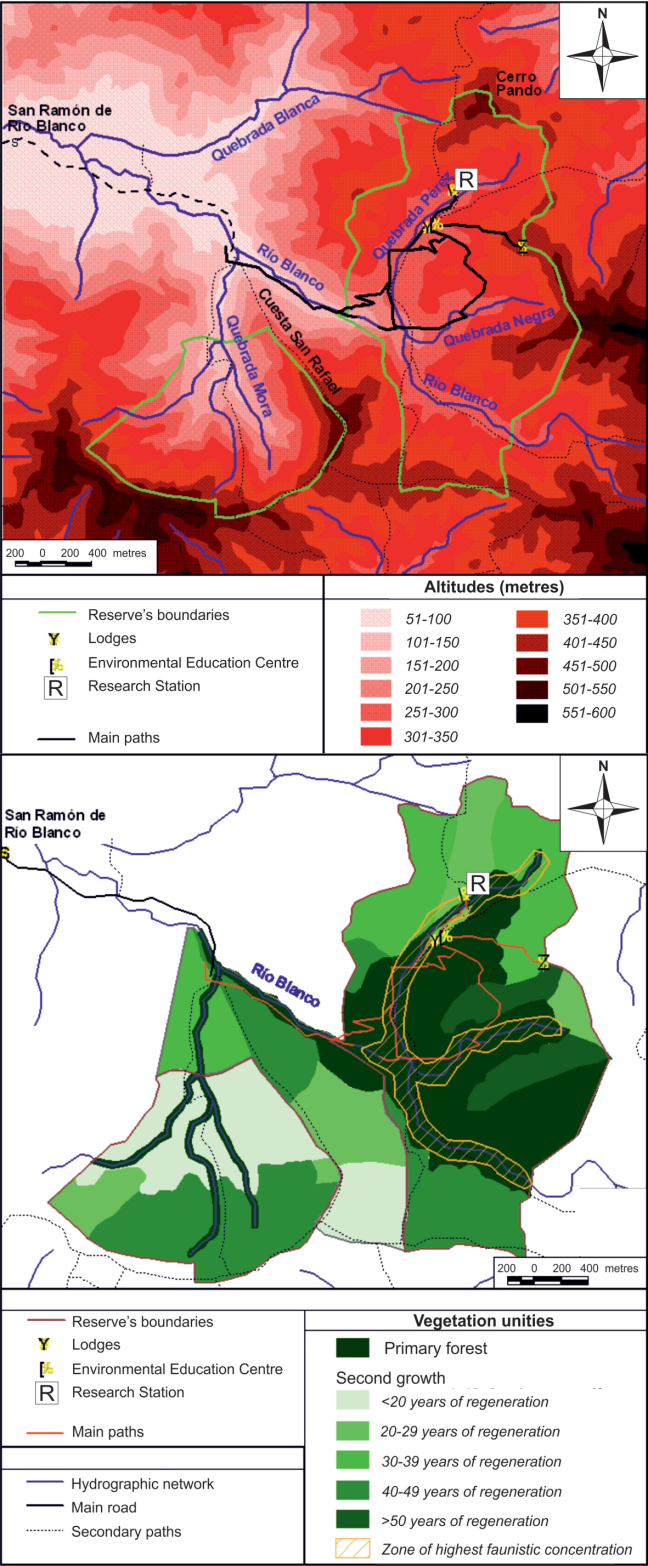
Innermost part of the Karen Mogensen Reserve, with altitudes (above), vegetation unities and their age (below), and hydrographic network shown.

**Figure 3.**
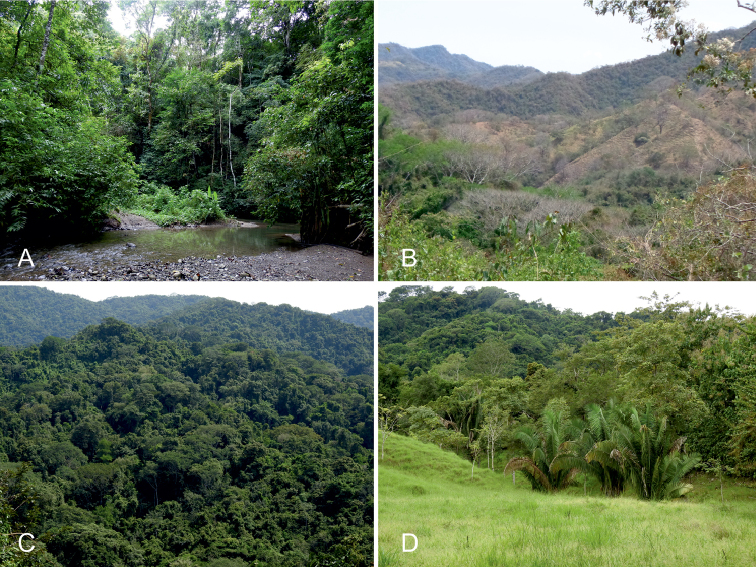
Main habitats of the Karen Mogensen Reserve. **A**, Moist forest **B** Dry forest **C** Dry to moist transitional forest **D** Forest borders and grassland. Photographs by: M. Dal Zotto (**A, C, D**) and G. Romeo (**B**).

The Karen Mogensen Reserve, placed in the SE Nicoya Peninsula, northwestern Costa Rica, is an inland foothill zone comprised between 9.85 and 9.88 degrees of latitude (N), and 85.04 and 85.08 degrees of longitude (W). It comprises approximately 1,000 hectares and shows an altitudinal range of almost 500 meters, from 130 to approximately 600 m a.s.l. (Figs 1, 2). Close to the Karen Mogensen Reserve is Cerro Pozo (maximum elevation 755 m a.s.l.), with its forested environments in direct continuity with those of the Reserve. The area is characterized mainly by a dry to moist transitional forest, and includes patches of pure dry and moist forest (Fig. 3; see the habitat classification by Holdridge 1967). Several rivers and streams created the growth conditions of a moist forest, contiguous with the widespread northwestern Costa Rican dry forest. In some parts of the area there are gallery forests. The interior part of the Karen Mogensen Reserve consists of primary forest, while the remaining is a second growth of different ages (20 to more than 50 years), partially resulting from conservation measures adopted from the 1990s which prompted the natural regeneration of the land previously used for cattle pastures and subsistence farming. Information on the age of the forest portions of the Karen Mogensen Reserve is shown in Fig. 2. The Karen Mogensen Reserve is currently a National Wildlife Refuge and part of the Biological Corridor of the Nicoya Peninsula. The lowest zones of the Karen Mogensen Reserve show a transition from forests to pastures and grassland. The protected area borders some cattle pastures and other diversely protected or unprotected second growth forested land, covering almost 12,000 hectares. The distances of the Karen Mogensen Reserve from the Gulf of Nicoya and from the open Pacific Ocean are approximately 10 km and approximately 15 km, respectively.

## Materials and methods

Since the foundation of the Karen Mogensen Reserve in 1996, nearly twenty ornithological surveys have been carried out, the latest of which ended in March 2017. The surveyed periods normally corresponded to the dry season or to the months characterized by lower precipitations (late October-late December and mid-January-beginning of May). We underline that long-term data sets from tropical regions collected under more rigorous protocols are not available from the majority of Neotropical study sites, for a number of reasons, as reported by other recent studies (see Boyle and Sigel 2015). However, in the present study, a more standardized data collection procedure was attempted in the last 12 surveys, carried out since 2005. The techniques adopted follow Bibby et al. (1998, 2000), Sutherland et al. (2004), and Woog et al. (2010). Bird registration was based on fixed points and transects, placed in order to sample different altitudes and all the main types of habitats. Environmental surveys to assess the altitudinal distribution and habitats of birds were based on Ferrer (2002) and Mendez and Mendez (2004), the latter relying on the classification by Holdridge (1967). A total of 13 fixed points were located at least 750 m from each other, and 13 transects established, each with an average length of 500 m, resulting in nearly 7 km of linear transects (Figure 1). Species diagnosis was based on direct observation or identification of vocalizations. Observations were made during ca. 10 minutes at each fixed point, often in alternating morning (6:00–09:00) and afternoon sampling periods (14:00–17:00). During the same hours, the transects were walked at an average speed of 1 km/h. The fixed points and transects were georeferenced with a GPS Garmin GPSMAP 64S (GPS accuracy: 3 m). The coordinates of fixed points, transects endpoints, altitudes, and habitats are reported in Table 1. The surveys normally lasted 5 to 15 days. This procedure resulted in mainly qualitative information. Several opportunistic observations or identification of vocalizations at night, the latest of which led until September 2017, were used to supplement the list of species. From November 2016 to March 2017, some individuals of mainly passerine birds were legally trapped using mist nets and then released, under a scientific investigation permit granted by the "Sistema Nacional de Areas de Conservación – Dirección Programa de Investigación, Area de Conservación Tempisque" (code: ACT-OR-DR-119-16). Species accumulation curves based on the last 12 surveys, led from 2005 to 2017, were created with the software PRIMER 6.0 (Clarke and Gorley 2006), under 1000 permutations and the Chao 1, Chao 2, first- and second-order Jackknife estimators.

**Table 1.** Transects endpoints and fixed points used for data collection. Coordinates, altitudes, and main habitats covered are reported. The numbers (transects endpoints) and Roman numerals (fixed points) refer to Fig. 1.

**Point**	**Latitude (N)**	**Longitude (W)**	**Altitude (m)**	**Habitat**
*Transects endpoints*				
1	9°51'41.443"N, 85°4'22.616"W		169	forest border
2	9°51'23.389"N, 85°4'17.476"W		209	second growth moist forest
3	9°51'49.331"N, 85°3'51.354"W		208	forest border
4	9°51'57.042"N, 85°3'37.429"W		303	primary moist forest
5	9°51'39.708"N, 85°3'34.978"W		281	primary moist forest
6	9°51'26.557"N, 85°3'30.434"W		302	primary moist forest
7	9°51'23.868"N, 85°3'21.470"W		304	primary moist forest
8	9°51'15.858"N, 85°3'7.722"W		322	primary moist forest
9	9°52'6.845"N, 85°3'31.392"W		314	primary moist forest
10	9°52'19.934"N, 85°3'19.199"W		344	primary transitional forest
11	9°52'15.571"N, 85°3'15.552"W		360	primary transitional forest
12	9°52'5.887"N, 85°3'2.880"W		441	second growth transitional forest
13	9°52'17.663"N, 85°3'27.983"W		333	second growth transitional forest
14	9°52'33.085"N, 85°3'25.711"W		419	second growth dry forest
15	9°52'23.282"N, 85°3'6.106"W		357	second growth transitional forest
16	9°52'22.922"N, 85°2'51.821"W		351	second growth dry forest
17	9°52'14.794"N, 85°2'44.171"W		338	second growth moist forest
18	9°51'57.402"N, 85°2'44.948"W		399	second growth moist forest
19	9°52'21.130"N, 85°2'41.482"W		323	second growth moist forest
20	9°52'26.152"N, 85°2'25.642"W		297	second growth transitional forest
21	9°52'39.241"N, 85°3'22.784"W		418	second growth dry forest
22	9°52'56.755"N, 85°3'20.632"W		388	second growth dry forest
23	9°52'49.343"N, 85°1'54.440"W		216	forest border
24	9°52'54.185"N, 85°1'35.731"W		204	second growth dry forest
25	9°52'5.250"N, 85°4'20.438"W		138	grassland
26	9°52'13.350"N, 85°4'37.826"W		134	grassland
*Fixed points*				
I	9°51'22.010"N, 85°4'16.738"W		191	second growth moist forest
II	9°51'49.097"N, 85°3'54.065"W		168	forest border
III	9°51'24.757"N, 85°3'26.129"W		302	primary moist forest
IV	9°52'2.154"N, 85°3'34.945"W		307	primary moist forest
V	9°52'20.734"N, 85°3'17.316"W		348	primary transitional forest
VI	9°52'4.746"N, 85°3'0.396"W		411	second growth dry forest
VII	9°51'41.875"N, 85°3'10.228"W		377	second growth transitional forest
VIII	9°52'20.734"N, 85°2'44.444"W		329	second growth transitional forest
IX	9°52'39.076"N, 85°2'21.340"W		270	second growth transitional forest
X	9°52'55.027"N, 85°1'33.020"W		180	forest border
XI	9°53'1.122"N, 85°3'22.064"W		369	second growth dry forest
XII	9°52'1.916"N, 85°4'19.247"W		143	grassland
XIII	9°52'15.254"N, 85°4'41.401"W		121	grassland

Information was collected on the horizontal and vertical distribution of the avian community. Species recorded in the same habitat were pooled to estimate habitat utilization. The habitat types were classified following Miller et al. (2015) in: (1) moist forest, (2) dry forest, (3) forest border, (4) grassland-pasture. The vertical distribution was based on grouping the species from the same forest height levels, in order to analyze community dynamics at different levels. The classification of forest height levels followed Levey and Stiles (1994) with some modifications: (1) understory (from ground level to 4 m), (2) middle level (main trees trunk), (3) canopy (uppermost level of the forest), (4) open air.

To characterize the resident avian community of the Karen Mogensen Reserve, waterbirds were excluded, i.e., members of the families Anatidae, Scolopacidae, Fregatidae, Ardeidae, Ciconiidae, and Threskiornithidae. Migratory and wintering birds were also excluded as well as a few occasional native species far from their normal geographic breeding distribution ranges. Thus, the typical resident bird community of the area was composed of breeding or potentially breeding species with mainly terrestrial habits. Bird breeding was ascertained by the direct observation of nests or through the collection of indirect evidence (e.g., presence of pulli, adults carrying material for nesting, adults in courtship, adults displaying territorial behavior during the breeding season). Each species was assigned to a forest dependency category, following Stiles (1985) and Sandoval and Barrantes (2009), namely: (1) species that live and reproduce in extensive mature forests, (2) species that inhabit habitats with 40–50% of forest cover, (3) species that live in open areas.

The taxonomic sequence and the scientific and common names of the registered taxa follow the AOU Checklist (American Ornithologists’ Union) through the 2016 supplement (Chesser et al. 2016) and the IOC World Bird List (v. 7.3) by Gill and Donsker (2017), with some modifications according to del Hoyo and Collar (2017) and del Hoyo et al. (2017c). The official checklists for Costa Rica considered for comparisons are those provided by Garrigues et al. (2015) and Lepage (2017). The information on the status of each species is derived from the IUCN database (last access: October 2017; IUCN 2017); other information on conservation aspects derives from Species+ database (UNEP 2017) accessed on 20 February 2017 and from the Red list provided by the Ornithological Association of Costa Rica (AOCR 2005). Additional features regarding the biology or the biogeography of some species were obtained from the Handbook of the birds of the world by del Hoyo et al. (1996, 1999, 2001, 2002, 2004), in some cases referring to the updated online version, and from the Global Biodiversity Information Facility (GBIF 2017).

## Data resources

The data reported in this paper is deposited at GBIF, the Global Biodiversity Information Facility, http://ipt.pensoft.net/resource.do?r=cr_karen_aves.

## Results

**Figure 4.**
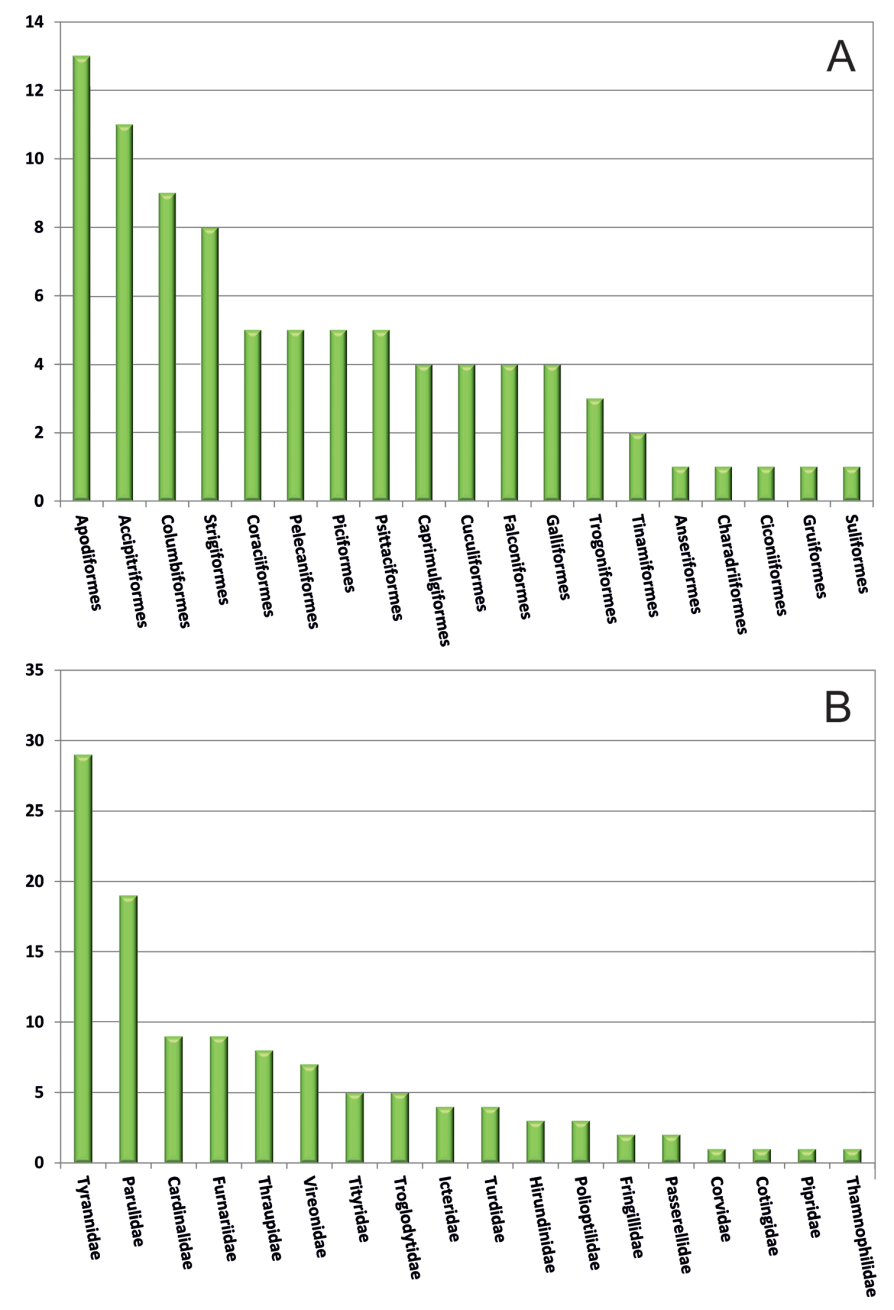
Avian richness of the Karen Mogensen Reserve. Histograms reporting the number of recorded species for each of the non-passerine orders (**A**) and of the passerine families (**B**).

**Figure 5.**
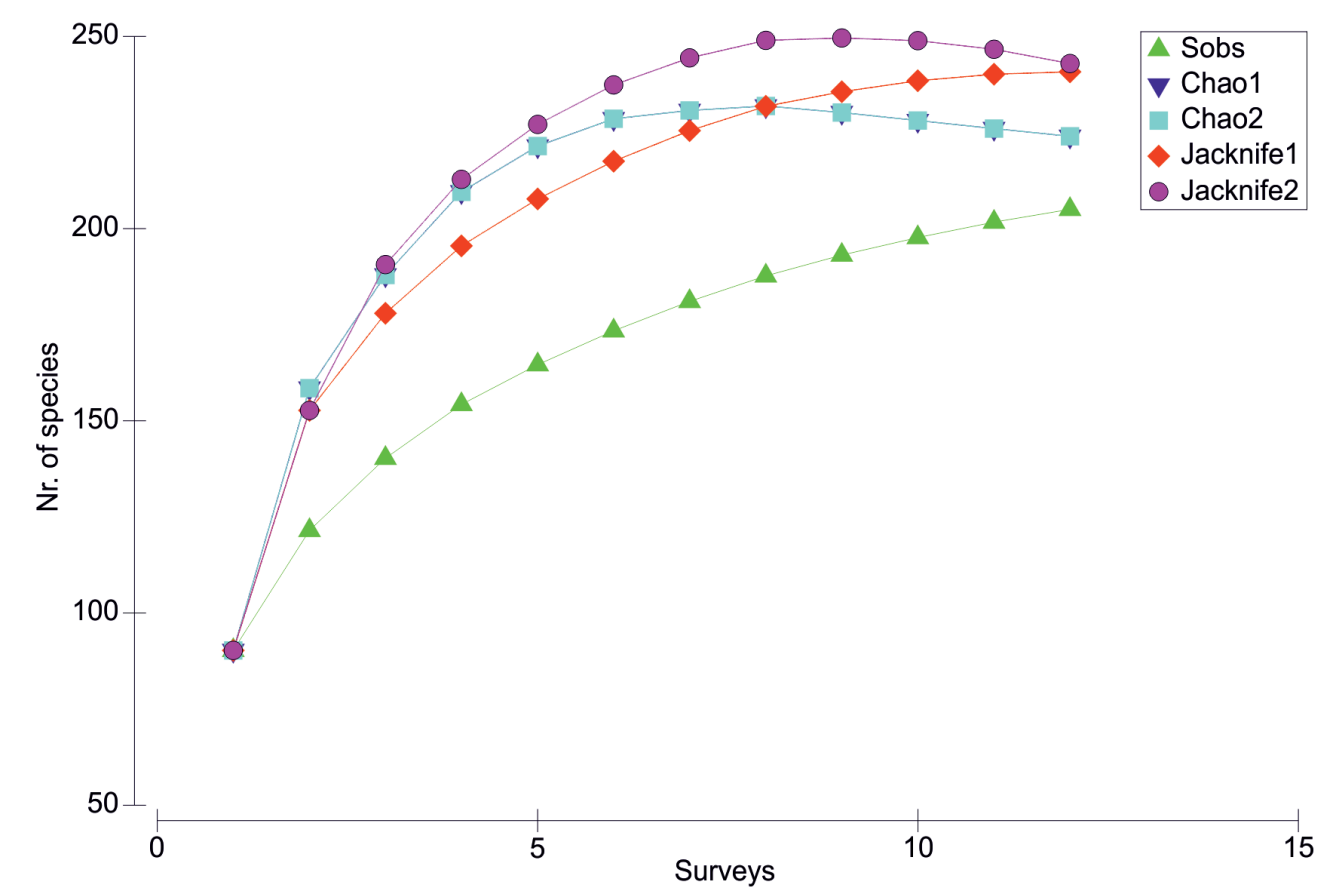
Species accumulation curves based on the surveys from 2005 to 2017. “Sobs” is the permuted observed species accumulation (1000 permutations).

**Figure 6.**
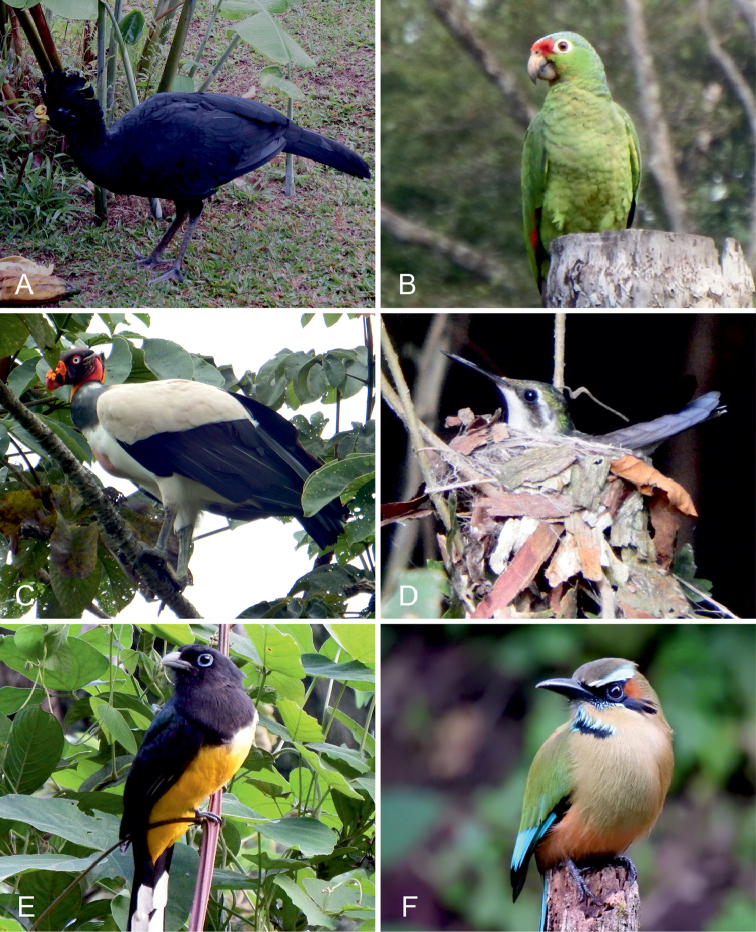
Non-passerine birds observed within the Karen Mogensen Reserve. **A** *Crax rubra* (Cracidae), a globally vulnerable species that reproduces in the area **B** *Amazona autumnalis* (Psittacidae), species resident and potentially breeding in the area, characterized by declining populations and considered threatened on a national scale **C** *Sarcoramphus papa* (Cathartidae), species resident in the area, with declining populations and considered locally threatened **D** *Chlorostilbon canivetii* (Trochilidae), female at nest, characterized by traits (e.g., the colour of the lower mandible) which resemble the congeneric *C. assimilis* **E** *Trogon melanocephalus* (Trogonidae) and **F** *Eumomota superciliosa* (Momotidae), two species that reach their southernmost range border approximately in the surveyed zone. Photographs by: M. Dal Zotto (**F**) and G. Romeo (**A, B, C, D, E**).

**Figure 7.**
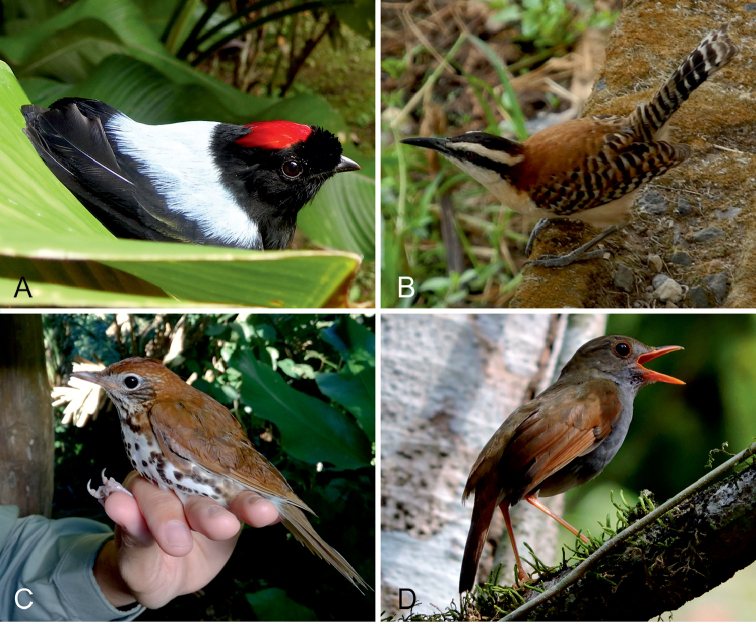
Passeriformes observed within the Karen Mogensen Reserve. **A** *Chiroxiphia linearis* (Pipridae), a species typical of Central American tropical dry forests reaching its southernmost range border in the area **B** *Campylorhynchus capistratus nicoyae* (Troglodytidae), a subspecies endemic to NW Costa Rica, which reaches its southernmost breeding areas in the surveyed zone **C** *Catharus aurantiirostris* (Turdidae), species characterized by a local isolated breeding population **D** *Hylocichla mustelina* (Turdidae), regular winter visitor in the Karen Mogensen Reserve, but never recorded before from the Nicoya Peninsula. Photographs by: M. Dal Zotto (**A, B, D**) and G. Romeo (**C**).

In total, 207 bird species in 153 genera, 47 families, and 23 orders were recorded (Table 2; Figs 4–7). The 22 orders of non-passerine birds account for 29 families, 75 genera, and 95 species, while the order Passeriformes includes 18 families, 78 genera, and 112 species. Tyrannidae was the most species rich family, with 29 species, followed by Parulidae (19 species), and Trochilidae (10 species; Fig. 4). These three families mainly included species that normally live in forested areas, being insect or nectar feeders, and that are chiefly resident (Trochilidae, Tyrannidae), or migrant or winter visitors from North America (Parulidae). Thirteen families included a single species each. It must be noted that five of these, namely Anatidae, Scolopacidae, Fregatidae, Ciconiidae, and Threskiornithidae, included water- or seabirds; these species were observed only occasionally in the mainly forested environment of the Karen Mogensen Reserve.

**Table 2.** Checklist of the birds (Aves) observed at the Karen Mogensen Reserve, Nicoya Peninsula, northwestern Costa Rica. The systematization, the scientific and common names follow the AOU Checklist (Chesser et al. 2016) and the IOC World Bird List v. 7.3 (Gill and Donsker 2017). Phenological and ecological categorizations follow Garrigues and Dean (2014) and Garrigues et al. (2015). Habitat types are classified following Miller et al. (2015). Height level classification follows Levey and Stiles (1994) with some modifications. Forest dependency categories follow Stiles (1985) and Sandoval and Barrantes (2009) with some modifications. Abbreviations. *Phenology*: **A**: accidental, **M**: migratory, **R**: resident, **RB**: resident breeding, **W**: wintering, **?**: doubtful phenology. *Habitat type*: **1**: moist forest, **2**: dry forest, **3**: forest border, **4**: grassland-pasture, **n.e.**: not evaluated. Height level: **1**: understory (from ground level to 4 m); **2**: middle level (main trees trunk); **3**: canopy (uppermost level of the forest); **4**: open air; **n.m.**: not measurable. *Forest dependency category*: **1**: species that lives and reproduces in extensive mature forest; **2**: species that inhabits habitats with 40–50% of forest cover; **3**: species that lives in open areas; **n.e.**, not evaluated.

Taxon	English name	**Phenology**	Habitat type	Height level	Forest Dependence category
TINAMIFORMES					
Tinamidae					
*Crypturellus cinnamomeus*	Thicket Tinamou	RB	1,2	1	1
*Crypturellus soui*	LittleTinamou	RB	1,2	1	1
ANSERIFORMES					
Anatidae					
*Cairina moschata*	Muscovy Duck	A	3,4	n.m.	n.e.
GALLIFORMES					
Cracidae					
*Crax rubra*	Great Curassow	RB	1,2	1	1
*Ortalis vetula*	Plain Chachalaca	RB	2,3	1,2	1,2
*Penelope purpurascens*	Crested Guan	RB	1,2	2,3	1
Odontophoridae					
*Colinus cristatus*	Crested Bobwhite	RB	4	1	3
COLUMBIFORMES					
Columbidae					
*Claravis pretiosa*	Blue Ground-Dove	R	3	1,2	n.e.
*Columbina inca*	Inca Dove	RB	4	1	2,3
*Columbina passerina*	Common Ground-Dove	RB	4	1	3
*Columbina talpacoti*	Ruddy Ground-Dove	RB	4	1	3
*Geotrygon montana*	Ruddy Quail-Dove	RB	4	1	n.e.
*Leptotila plumbeiceps*	Gray-headed Dove	R	1	1	n.e.
*Leptotila verreauxi*	White-tipped Dove	RB	1,2,3,4	1	1,2,3
*Patagioenas flavirostris*	Red-billed Pigeon	RB	2,3,4	2,3	2,3
*Zenaida asiatica*	White-winged Dove	RB?	2,3,4	2,3	2,3
CUCULIFORMES					
Cuculidae					
*Crotophaga sulcirostris*	Groove-billed Ani	RB	3,4	1,2	3
*Morococcyx erythropygus*	Lesser Ground-Cuckoo	R	2,3,4	1,2	n.e.
*Piaya cayana*	Squirrel Cuckoo	RB	1,2,3	2,3	1,2
*Tapera naevia*	Striped Cuckoo	RB	1,3	1,2	2
CAPRIMULGIFORMES					
Caprimulgidae					
*Chordeiles acutipennis*	Lesser Nighthawk	M	3,4	1,4	n.e.
*Chordeiles minor*	Common Nighthawk	M	3,4	1,4	n.e.
*Nyctidromus albicollis*	Common Pauraque	RB	1,2,3,4	1	1,2
NYCTIBIIFORMES					
Nyctibiidae					
*Nyctibius jamaicensis*	Northern Potoo	RB	2,3,4	2	2,3
APODIFORMES					
Apodidae					
*Chaetura vauxi*	Vaux’s Swift	R	1,2,3,4	4	n.e.
*Panyptila cayennensis*	Lesser Swallow-tailed Swift	RB	1	4	1,2,3
*Streptoprocne zonaris*	White-collared Swift	R	1,2,3,4	4	n.e.
Trochilidae					
*Amazilia rutila*	Cinnamon Hummingbird	RB	2,3	1	2
*Amazilia saucerottei*	Steely-vented Hummingbird	RB	1,2,3	1,2	1,2
*Amazilia tzacatl*	Rufous-tailed Hummingbird	RB	1,2,3	1,2	1,2
*Anthracothorax prevostii*	Green-breasted Mango	RB	1,2,3	1,2	1,2
*Archilochus colubris*	Ruby-throated Hummingbird	M, W	1,2,3	1,2	n.e.
*Chlorostilbon canivetii*	Canivet’s Emerald	RB	1,3	1,2	2
*Heliomaster constantii*	Plain-capped Starthroat	RB	2,3,4	2,3	2,3
*Hylocharis eliciae*	Blue-throated Goldentail	RB	1,2,3	1,2	1,2
*Phaeochroa cuvierii*	Scaly-breasted Hummingbird	RB?	1,2,3	1,2	1,2
*Phaethornis striigularis*	Stripe-throated Hermit	RB	1,3	1	1,2
GRUIFORMES					
Rallidae					
*Aramides cajaneus*	Gray-cowled Wood-Rail	RB	1,2,3	1	1,2
CHARADRIIFORMES					
Scolopacidae					
*Actitis macularius*	Spotted Sandpiper	A	n.e.	n.m.	n.e.
CICONIIFORMES					
Ciconiidae					
*Mycteria americana*	Wood Stork	A	4	1,2	n.e.
SULIFORMES					
Fregatidae					
*Fregata magnificens*	Magnificent Frigatebird	A	n.e.	n.m.	n.e.
PELECANIFORMES					
Ardeidae					
*Ardea alba*	Great Egret	M	3	1	n.e.
*Bubulcus ibis*	Cattle Egret	M	4	1	n.e.
*Butorides virescens*	Green Heron	M	1,2,3,4	1	n.e.
*Cochlearius cochlearius*	Boat-billed Heron	M	3,4	1,2	n.e.
*Egretta caerulea*	Little Blue Heron	M	3,4	1	n.e.
*Tigrisoma mexicanum*	Bare-throated Tiger-Heron	RB	1,3,4	1	1,2,3
Threskiornithidae					
*Eudocimus albus*	White Ibis	A	n.e.	n.m.	n.e.
CATHARTIFORMES					
Cathartidae					
*Cathartes aura*	Turkey Vulture	RB	1,2,3,4	3,4	2,3
*Coragyps atratus*	Black Vulture	RB	1,2,3,4	3,4	2,3
*Sarcoramphus papa*	King Vulture	R	1,2	3,4	n.e.
ACCIPITRIFORMES					
Pandionidae					
*Pandion haliaetus*	Osprey	A	n.e.	4	n.e.
Accipitridae					
*Buteo albonotatus*	Zone-tailed Hawk	M	4	4	n.e.
*Buteo brachyurus*	Short-tailed Hawk	M	1,2,3	4	n.e.
*Buteo plagiatus*	Gray Hawk	RB?	3,4	2,3	2,3
*Buteo platypterus*	Broad-winged Hawk	M, W	1,2,3	2,4	n.e.
*Buteo swainsonii*	Swainson’s Hawk	A	4	4	n.e.
*Buteogallus anthracinus*	Common Black Hawk	A	1,2,3	2,4	n.e.
*Ictinia plumbea*	Plumbeous Kite	M	1	3,4	n.e.
*Leptodon cayanensis*	Gray-headed Kite	RB?	1,2,3	3	1,2
*Morphnarchus princeps*	Barred Hawk	A	1,3	2,4	n.e.
*Pseudastur albicollis*	White Hawk	RB	1,3	2,4	1,2
*Rupornis magnirostris*	Roadside Hawk	RB	1,2,3	1,4	2,3
STRIGIFORMES					
Tytonidae					
*Tyto alba*	Barn Owl	A	3,4	1,2	n.e.
Strigidae					
*Ciccaba nigrolineata*	Black-and-white Owl	A	1,3	2,3	n.e.
*Ciccaba virgata*	Mottled Owl	RB	1,2,3	2	1,2
*Glaucidium brasilianum*	Ferruginous Pygmy-Owl	RB	1,2,3	2,3	1,2
*Lophostrix cristata*	Crested Owl	RB	1,2,3	1,2	1,2
*Megascops choliba*	Tropical Screech-Owl	RB?	3	1,2	2
*Megascops cooperi*	Pacific Screech-Owl	RB	2,3,4	1,2	1,2,3
*Megascops guatemalae*	Vermiculated Screech-Owl	RB	1	1,2	1
*Pulsatrix perspicillata*	Spectacled Owl	RB	1,2	2,3	1
TROGONIFORMES					
Trogonidae					
*Trogon caligatus*	Gartered Trogon	RB	1,3	2,3	1,2
*Trogon elegans*	Elegant Trogon	RB	1,2,3	2,3	1,2
*Trogon melanocephalus*	Black-headed Trogon	RB	1,2,3	1,2	1,2
CORACIIFORMES					
Momotidae					
*Eumomota superciliosa*	Turquoise-browed Motmot	RB	1,2,3	1,2	1,2
*Momotus coeruliceps*	Blue-capped Motmot	RB	2,3,4	1,2	1,2,3
Alcedinidae					
*Megaceryle torquata*	Ringed Kingfisher	R	1,3	2	n.e.
*Chloroceryle amazona*	Amazon Kingfisher	R	1,3	2	n.e.
*Chloroceryle americana*	Green Kingfisher	RB	1,3	1	2
PICIFORMES					
Bucconidae					
*Notharchus hyperrhynchus*	White-necked Puffbird	RB	1,2,3	3	1,2
Ramphastidae					
*Pteroglossus torquatus*	Collared Aracari	RB	1,3	2,3	1,2
Picidae					
*Campephilus guatemalensis*	Pale-billed Woodpecker	RB	1,2,3	1,2,3	1,2
*Dryocopus lineatus*	Lineated Woodpecker	RB	1,2,3,4	1,2,3	2
*Melanerpes hoffmannii*	Hoffmann’s Woodpecker	RB	2,3,4	2,3	1,2,3
FALCONIFORMES					
Falconidae					
*Caracara cheriway*	Crested Caracara	RB?	2,3,4	1,3	2,3
*Falco rufigularis*	Bat Falcon	RB	1,3	3,4	1,2
*Herpetotheres cachinnans*	Laughing Falcon	RB	1,2,3	2,3	1,2,3
*Micrastur semitorquatus*	Collared Forest-Falcon	RB	1,2,3	2	1
PSITTACIFORMES					
Psittacidae					
*Amazona albifrons*	White-fronted Amazon	RB	2,3	2,3	1,2
*Amazona auropalliata*	Yellow-naped Amazon	R	2,3,4	2,3	2,3
*Amazona autumnalis*	Red-lored Amazon	RB	1,3	3	1,2
*Brotogeris jugularis*	Orange-chinned Parakeet	RB	1,2,3	3	2
*Eupsittula canicularis*	Orange-fronted Parakeet	RB	2,3	3	2
PASSERIFORMES					
Thamnophilidae					
*Thamnophilus doliatus*	Barred Antshrike	RB	1,2,3	1	1,2
Furnariidae					
*Dendrocincla fuliginosa*	Plain-brown Woodcreeper	RB?	1	1,2	1
*Dendrocincla homochroa*	Ruddy Woodcreeper	RB	1,2	1,2	1
*Dendrocolaptes sanctithomae*	Northern Barred-Woodcreeper	RB	1,2,3	1,2	1,2
*Glyphorynchus spirurus*	Wedge-billed Woodcreeper	RB?	1	1,2	1
*Lepidocolaptes souleyetii*	Streak-headed Woodcreeper	RB	1,2,3	2	1,2
*Sittasomus griseicapillus*	Olivaceous Woodcreeper	RB	1,2,3	2	1,2
*Xenops minutus*	Plain Xenops	RB	1,2,3	1,2	1,2
*Xiphorhynchus flavigaster*	Ivory-billed Woodcreeper	RB	2,3	1,2	1,2
*Xiphorhynchus susurrans*	Cocoa Woodcreeper	A	1,2,3	1,2,3	1,2
Tyrannidae					
*Attila spadiceus*	Bright-rumped Attila	RB	1,2,3	1,2	1,2
*Camptostoma imberbe*	Northern Beardless-Tyrannulet	A	2,3	2,3	n.e.
*Contopus sordidulus*	Western Wood-Pewee	A	1,2,3	1,2	n.e.
*Contopus virens*	Eastern Wood-Pewee	M	1,2,3	1,2	n.e.
*Elaenia flavogaster*	Yellow-bellied Elaenia	RB	3	1,2	2
*Empidonax flaviventris*	Yellow-bellied Flycatcher	M	1,2,3	1,2	n.e.
*Empidonax traillii*	Willow Flycatcher	M, W	3	1	n.e.
*Legatus leucophaius*	Piratic Flycatcher	A	3	3	n.e.
*Megarhynchus pitangua*	Boat-billed Flycatcher	RB	1,2,3	2,3	1,2
*Mionectes oleagineus*	Ochre-bellied Flycatcher	RB	1,3	1,2	1,2
*Myiarchus crinitus*	Great Crested Flycatcher	M, W	1,2,3	2,3	n.e.
*Myiarchus nuttingi*	Nutting’s Flycatcher	RB	2,3	1,2	1,2
*Myiarchus tuberculifer*	Dusky-capped Flycatcher	RB	3	1,2	2
*Myiarchus tyrannulus*	Brown-crested Flycatcher	RB	2,3,4	1,2	2,3
*Myiodynastes luteiventris*	Sulphur-bellied Flycatcher	A	1,2,3	2,3	n.e.
*Myiodynastes maculatus*	Streaked Flycatcher	RB	1,2,3	2,3	1,2,3
*Myiopagis viridicata*	Greenish Elaenia	RB	1,2,3	1,2	1,2
*Myiozetetes similis*	Social Flycatcher	RB	1,2,3	2,3	2
*Oncostoma cinereigulare*	Northern Bentbill	RB	1,2	1,2	1,2
*Onychorhynchus coronatus*	Royal Flycatcher	RB	1,2,3	1,2	1,2
*Pitangus sulphuratus*	Great Kiskadee	RB	1,2,3,4	2	2,3
*Platyrinchus cancrominus*	Stub-tailed Spadebill	RB	1,2	1	1,2
*Poecilotriccus sylvia*	Slate-headed Tody-Flycatcher	RB	1,2	1	1,2
*Todirostrum cinereum*	Common Tody-Flycatcher	RB	1,2,3	1,2	1,2
*Tolmomyias sulphurescens*	Yellow-olive Flycatcher	RB	1,2,3	1,2	1,2
*Tyrannus forficatus*	Scissor-tailed Flycatcher	M, W	3,4	2	n.e.
*Tyrannus melancholicus*	Tropical Kingbird	RB	3,4	2	3
*Tyrannus verticalis*	Western Kingbird	M, W	2,3,4	2	n.e.
*Zimmerius vilissimus*	Paltry Tyrannulet	RB	1,2,3	2,3	2
Tityridae					
*Pachyramphus aglaiae*	Rose-throated Becard	RB	1,2,3	2,3	1,2
*Pachyramphus cinnamomeus*	Cinnamon Becard	RB?	3	1,2	2
*Pachyramphus polychopterus*	White-winged Becard	RB	1,3	2,3	1,2
*Tityra inquisitor*	Black-crowned Tityra	RB?	1,2,3	3	1,2
*Tityra semifasciata*	Masked Tityra	RB	1,2,3	3	1,2
Cotingidae					
*Procnias tricarunculatus*	Three-wattled Bellbird	M	1	2,3	n.e.
Pipridae					
*Chiroxiphia linearis*	Long-tailed Manakin	RB	1,2,3	1,2	1,2
Vireonidae					
*Cyclarhis gujanensis*	Rufous-browed Peppershrike	RB	1,2,3	2,3	1,2
*Pachysylvia decurtata*	Lesser Greenlet	RB	1,2,3	2,3	1,2
*Vireo flavifrons*	Yellow-throated Vireo	M, W	1,2,3	2	n.e.
*Vireo flavoviridis*	Yellow-green Vireo	A	1,2,3	2	n.e.
*Vireo olivaceus*	Red-eyed Vireo	M	1,2,3	2,3	n.e.
*Vireo philadelphicus*	Philadelphia Vireo	M, W	1,2,3	2,3	n.e.
*Vireo solitarius*	Blue-headed Vireo	M, W	1,2,3	2,3	n.e.
Corvidae					
*Cyanocorax formosus*	White-throated Magpie-Jay	RB	2,3,4	2	2
Hirundinidae					
*Hirundo rustica*	Barn Swallow	M	4	4	n.e.
*Petrochelidon pyrrhonota*	Cliff Swallow	M, W?	4	4	n.e.
*Progne chalybea*	Gray-breasted Martin	RB	4	4	3
Troglodytidae					
*Campylorhynchus capistratus*	Rufous-naped Wren	RB	2,3	2,3	1,2
*Cantorchilus modestus*	Cabanis’s Wren	RB	3	1	2
*Thryophilus pleurostictus*	Banded Wren	RB	1,2,3	1	1,2
*Thryophilus rufalbus*	Rufous-and-white Wren	RB	1,2,3	1,2	1,2
*Troglodytes aedon*	House Wren	RB?	3,4	1	2
Polioptilidae					
*Polioptila albiloris*	White-lored Gnatcatcher	RB	1,2,3,4	1	2
*Polioptila plumbea*	Tropical Gnatcatcher	RB	1,2,3	2,3	1,2
*Ramphocaenus melanurus*	Long-billed Gnatwren	RB	1,2,3	1,2	1,2
Turdidae					
*Catharus aurantiirostris*	Orange-billed Nightingale-Thrush	RB	1,3	1	1,2
*Catharus ustulatus*	Swainson’s Thrush	M	1,2,3	1,2	n.e.
*Hylocichla mustelina*	Wood Thrush	M, W?	1,2,3	1	n.e.
*Turdus grayi*	Clay-colored Thrush	RB	1,2,3	1,2	2
Fringillidae					
*Euphonia affinis*	Scrub Euphonia	RB	2,3	2,3	1,2
*Euphonia hirundinacea*	Yellow-throated Euphonia	RB	1,2,3	2,3	1,2
Parulidae					
*Basileuterus rufifrons*	Rufous-capped Warbler	RB	1,2,3	1,2	1,2
*Cardellina pusilla*	Wilson’s Warbler	M, W	1,2,3	1,2,3	n.e.
*Geothlypis formosa*	Kentucky Warbler	M, W	1,2,3	1	n.e.
*Geothlypis poliocephala*	Gray-crowned Yellowthroat	RB?	4	1	3
*Geothlypis trichas*	Common Yellowthroat	M	4	1	n.e.
*Helmitheros vermivorum*	Worm-eating Warbler	M	1,2	1,2	n.e.
*Mniotilta varia*	Black-and-white Warbler	M, W	1,2,3	2,3	n.e.
*Myiothlypis fulvicauda*	Buff-rumped Warbler	RB?	2,3	1	1,2
*Oreothlypis peregrina*	Tennessee Warbler	M, W	3	1,2,3	n.e.
*Parkesia motacilla*	Louisiana Waterthrush	M	1	1	n.e.
*Parkesia noveboracensis*	Northern Waterthrush	M, W	1,3	1	n.e.
*Protonotaria citrea*	Prothonotary Warbler	M, W?	1,3	1	n.e.
*Seiurus aurocapilla*	Ovenbird	M, W	1,2,3	1	n.e.
*Setophaga fusca*	Blackburnian Warbler	M	1,3	2,3	n.e.
*Setophaga magnolia*	Magnolia Warbler	M, W	1,3	2	n.e.
*Setophaga pensylvanica*	Chestnut-sided Warbler	M, W	1,3	1,2	n.e.
*Setophaga petechia*	Yellow Warbler	A	3	1	n.e.
*Setophaga ruticilla*	American Redstart	M	1,2,3	1,2,3	n.e.
*Setophaga virens*	Black-throated Green Warbler	M, W	1,3	2,3	n.e.
Thraupidae					
*Cyanerpes cyaneus*	Red-legged Honeycreeper	RB	1,2,3	2,3	1,2
*Eucometis penicillata*	Gray-headed Tanager	RB	1,2,3	1	1,2
*Saltator coerulescens*	Grayish Saltator	RB	3	1	2
*Saltator maximus*	Buff-throated Saltator	RB	3	2	2
*Sporophila torqueola*	White-collared Seedeater	RB	4	1	3
*Thraupis episcopus*	Blue-gray Tanager	RB	1,2,3,4	2	1,2,3
*Tiaris olivaceus*	Yellow-faced Grassquit	RB?	4	1	3
*Volatinia jacarina*	Blue-black Grassquit	RB	4	1	3
Passerellidae					
*Arremonops rufivirgatus*	Olive Sparrow	RB	2,3	1	1,2
*Peucaea ruficauda*	Stripe-headed Sparrow	RB	3,4	1	2,3
Cardinalidae					
*Cyanocompsa cyanoides*	Blue-black Grosbeak	RB	1,3	1	1,2
*Habia rubica*	Red-crowned Ant-Tanager	RB	1,2	1	1
*Passerina caerulea*	Blue Grosbeak	RB?	2,3,4	1	2,3
*Passerina ciris*	Painted Bunting	M	1,2,3,4	1	n.e.
*Passerina cyanea*	Indigo Bunting	M, W	4	1	n.e.
*Pheucticus ludovicianus*	Rose-breasted Grosbeak	M, W	3	1,2	n.e.
*Piranga ludoviciana*	Western Tanager	M, W	1,2,3	2,3	n.e.
*Piranga olivacea*	Scarlet Tanager	M	1,2,3	2,3	n.e.
*Piranga rubra*	Summer Tanager	M, W	1,2,3	2,3	n.e.
Icteridae					
*Amblycercus holosericeus*	Yellow-billed Cacique	RB	1,2,3	1	1,2
*Icterus galbula*	Baltimore Oriole	M, W	3	2,3	n.e.
*Icterus pustulatus*	Streak-backed Oriole	RB	2,3	2,3	1,2
*Molothrus bonariensis*	Shiny Cowbird	A	3,4	1,2	n.e.

The species accumulation curve based on the surveys led from 2005 to 2017 tended to stabilize, providing an indication of the completeness of the investigations. Plotted values for Chao 1 and 2, Jackknife 1 and 2 estimators were higher than the species richness observed (Fig. 5). The mist-netting activity led from November 2016 to March 2017 allowed to capture over 60 species of mainly passerine birds, one of which, *Vireo flavoviridis* (Vireonidae), was not previously recorded in the Karen Mogensen Reserve. In addition, individuals of species observed only occasionally were captured (e.g., the tyrant flycatcher *Myiodynastes luteiventris*). The capture of *Dendrocincla fuliginosa* (Furnariidae) was interesting as well, because it provided information on a species considered only potentially breeding in the Karen Mogensen Reserve. The capture of the following species was relevant nonetheless for different reasons: *Hylocichla mustelina* (Turdidae) and *Passerina ciris* (Cardinalidae), threatenend at a global scale, *Campylorhynchus capistratus*, present with a subspecies limited to the Nicoya Peninsula (*C. capistratus nicoyae*; see below), and *Catharus aurantiirostris*, characterized by a local breeding population (see below for details). Eventually, the use of mist-nets allowed to collect data on species rarely observed, namely: *Xenops minutus* and *Dendrocincla homochroa* (Furnariidae), *Tolmomyias sulphurescens*, *Mionectes oleagineus* and *Platyrinchus cancrominus* (Tyrannidae), *Ramphocaenus melanurus* (Polioptilidae), *Protonotaria citrea* (Parulidae).

**Figure 8.**
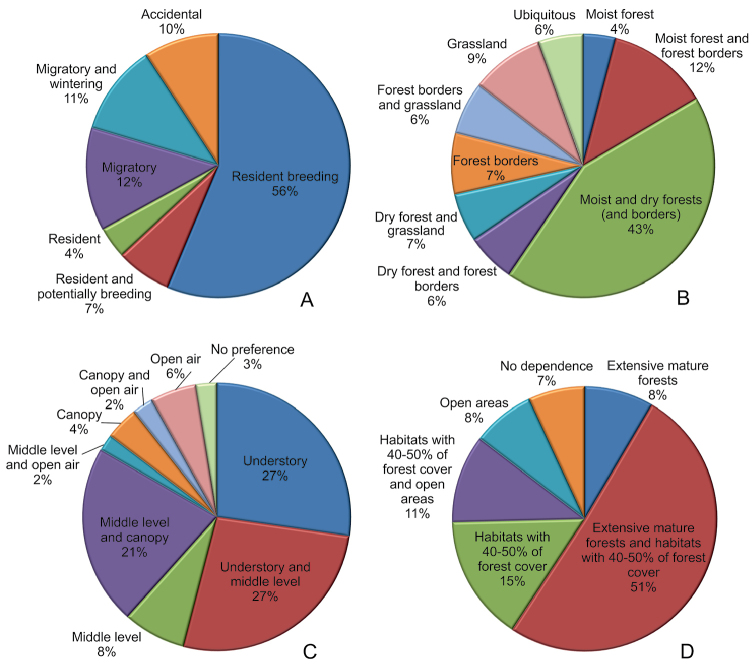
Ecological characterization of the avian community of the Karen Mogensen Reserve. Percentage of the recorded species based on: **A** Phenology **B** Habitat type **C** Height level **D** Forest dependency category.

Overall, 138 species were documented (70 non-passerines and 68 Passeriformes) that were resident in the Karen Mogensen Reserve and/or in the surrounding areas. Of these, 115 species (56 non-passerines and 59 Passeriformes) were breeding in the surveyed area (not always regularly), while another 14 species (six non-passerines and eight Passeriformes) were potentially breeding in the protected zone or, likely, in the adjacent forested land, as they were observed during their breeding season and showed territorial behavior. Eight species were resident but not breeding in the area. Forty-nine species were migratory; 23 of these also spent the winter in the Karen Mogensen Reserve, and another three were potentially wintering species. The remaining 20 species have been recorded one to ten times in the study period, thus were considered accidental in the Karen Mogensen Reserve (Fig. 8A).

Two species, i.e., *Melanerpes hoffmannii* (Picidae) and *Procnias tricarunculatus* (Cotingidae), are endemic to an area ranging from southern Honduras to Panama. *M. hoffmannii* was found breeding in the area, whereas *P. tricarunculatus* was observed only irregularly. In addition, we report the presence and breeding of four subspecies endemic to northwestern Costa Rica, namely: *Crypturellus cinnamomeus praepes* (Tinamidae), *Chlorostilbon canivetii salvini* (Trochilidae), *Xiphorhynchus flavigaster ultimus* (Furnariidae) and *Campylorhynchus capistratus nicoyae* (Troglodytidae; Fig. 7B). Furthermore, the presence of isolated breeding populations of *Ortalis vetula* (Cracidae) and *Catharus aurantiirostris* were detected (Turdidae; Fig. 7D; see below for further information).

Interestingly, 26 mainly resident breeding or potentially breeding species reach their global southernmost range border within the Nicoya Peninsula or the adjacent areas, i.e., *Crypturellus cinnamomeus* (Tinamidae), *Ortalis vetula* (Cracidae), *Columbina inca* (Columbidae), *Morococcyx erythropygus* (Cuculidae), *Nyctibius jamaicensis* (Nyctibiidae), *Amazilia rutila* and *Chlorostilbon canivetii* (Trochilidae), *Buteo plagiatus* (Accipitridae), *Megascops cooperi* (Strigidae), *Trogon elegans* and *T. melanocephalus* (Trogonidae; Fig. 6E), *Eumomota superciliosa* (Momotidae; Fig. 6F), *Melanerpes hoffmannii* (Picidae), *Eupsittula canicularis* and *Amazona albifrons* (Psittacidae), *Xiphorhynchus flavigaster* (Furnariidae), *Chiroxiphia linearis* (Pipridae; Fig. 7A), *Cyanocorax formosus* (Corvidae), *Polioptila albiloris* (Polioptilidae), *Campylorhynchus capistratus* (Fig. 7B) and *Thryophilus pleurostictus* (Troglodytidae), *Euphonia affinis* (Fringillidae), *Arremonops rufivirgatus* and *Peucaea ruficauda* (Passerellidae), *Passerina caerulea* (Cardinalidae), and *Icterus pustulatus* (Icteridae). In addition, a migratory species, *Piranga ludoviciana* (Thraupidae), reaches its southernmost wintering area within the Peninsula, and a second species, *Amazona auropalliata* (Psittacidae), resulted resident in the past but has been reported only occasionally in recent years.

Focusing on the habitat type, eight species were strictly connected to moist forest environments, and six of them potentially reproduced in the area. Another 24 species were associated to moist forests and forest borders. Eighty-seven species were related to either moist or dry forests, and, in some cases, also forest borders. None of the registered species was strictly associated to dry forests, but 12 taxa were associated to dry forests and their borders, and 13 species were associated to dry forests, forest borders, and grassland. Fifteen species were associated to forest borders, and 18 to grassland only (Fig. 8B). Thirteen species were typically associated to both forest edges and grassland. Eventually, eleven species did not show any preference for specific habitats, and can be found from moist forests to grasslands. To sum up this information, as expected a priori, the majority (65%) of the registered species is strictly connected to a forested environment: 32 to moist forests and their borders, 12 to dry forest environments and their edges, and 86 to either moist or dry forests (Fig. 8B).

Regarding vertical distribution (tree height level), 54 species were associated to the understory, 53 to either the understory and middle tree level, 15 to middle level only, 42 to both middle level and canopy, nine to canopy, four to middle level and open air, five to canopy and open air, eleven to open air, and only six species show no preference, being found from understory to canopy (Fig. 8C).

Of 129 resident breeding or potentially breeding species, eleven species were found and reproduced only in extensive mature forests, 20 in habitats with 40–50% of forest cover, 65 in both extensive mature forests and in habitats with 40–50% of forest cover, ten in open areas, 14 in both open areas and habitats with 40–50% of forest cover, and nine appeared to be not specifically associated to any of the three categories (Fig. 8D).

Five species that are on the Red List of globally threatened bird species (IUCN 2017) were detected, comprising three species classified as Vulnerable (*Crax rubra*, *Amazona auropalliata* and *Procnias tricarunculatus*), and two species classified as Near Threatened (*Hylocichla mustelina* and *Passerina ciris*). *C. rubra* was a resident breeder in the Karen Mogensen Reserve (Fig. 6A), whereas *H. mustelina* (Fig. 7C), and *P. ciris* were regular winter visitors, and *P. tricarunculatus* was observed only occasionally in the area during the winter season. *A. auropalliata* was resident in the first years after the establishment of the Karen Mogensen Reserve but has been observed only occasionally during the last surveys (see below). Another eight species are considered threatened at the national level and are included in the Costa Rican red list (AOCR 2005). These species are characterized by declining populations, namely: *Cairina moschata*, *Penelope purpurascens*, *Sarcoramphus papa* (Fig. 6C), *Lophostrix cristata*, *Falco rufigularis*, *Amazona autumnalis* (Fig. 6B), *Brotogeris jugularis*, and *Eupsittula canicularis*. More generally, this red list reports all the species included in Appendix II of CITES (UNEP 2017), thus all the 36 (overall) members of the families: Trochilidae (ten spp.), Pandionidae (one sp.), Accipitridae (eleven spp.), Falconidae (four spp.), Tytonidae (one sp.), and Strigidae (eight spp.). Summarizing, approximately one fourth (49 species altogether) of the total number of species recorded in the Karen Mogensen Reserve is considered significant in terms of bird conservation at a global or national level.

Based on the range maps by Garrigues and Dean (2014) and del Hoyo et al. (1996, 1999, 2001, 2002, 2004, plus online update), six species previously unknown from the Nicoya Peninsula were actually present within the Karen Mogensen Reserve, and represent range extensions of at least 100 km. These are: *Geotrygon montana* (Columbidae), *Panyptila cayennensis* (Apodidae), *Ciccaba nigrolineata* (Strigidae), *Xiphorhynchus susurrans* (Furnariidae), *Hylocichla mustelina* (Turdidae), and *Molothrus bonariensis* (Icteridae). Two of these, viz. *G. montana* and *P. cayennensis*, were found breeding in the area, and another one, *H. mustelina*, was a regular winter visitor. The presence of *C. nigrolineata* and *Xiphorhynchus susurrans* was detected in some occasions only during the last ornithological surveys (November 2016–March 2017), hence these species were considered accidental in the protected zone. We highlight that the icterid *M. bonariensis* is reported for the first time from the northwestern Pacific slope of Costa Rica (see below for additional details). Eventually, another species present in the area, *Tiaris olivaceus* (Thraupidae), was not reported in range maps prior to this study (e.g., Garrigues and Dean 2014), although previous records by MacArthur (1972), Villareal Orias et al. (2003), and Rising (2017) were known.

In the following accounts details on eight species of particular taxonomic, biogeographic, and/or conservational interest are provided.

### Accounts on species of interest

**Great Curassow** (*Crax rubra* Linnaeus, 1758; Fig. 6A)

Great Curassow is considered globally Vulnerable (IUCN 2017) and listed in the Appendix III of CITES (UNEP 2017). The species has undergone a rapid decline due to a high hunting pressure and habitat loss and fragmentation (BirdLife International 2016a). Although widespread in Costa Rica, it is observed mainly in protected areas and results uncommon to rare in the northwestern part of the country, where a reintroduction project has taken place recently (Zepeda 2006). Until the 1990s the distribution of this species in the Nicoya Peninsula was limited to the Cabo Blanco Absolute Natural Reserve, at the southernmost point of the peninsula. In the last decade, due to the natural forest regeneration, *C. rubra* has reached the Karen Mogensen Reserve, where it was historically known to occur. Several direct observations of isolated adult individuals were made, frequently during feeding activity on *Manilkara chicle* fruits. The species reproduced in the surveyed zone with an estimated three to four pairs. Further investigations are needed to verify the size of the local population in suitable habitats of the Karen Mogensen Reserve. This knowledge is the basis for the development of local conservation actions aimed at widening land protection and reducing hunting as suggested by BirdLife International (2016a).

**Plain Chachalaca** (*Ortalis vetula* [Wagler, 1830])

This species was often associated to dry forests or shrub covering the foothills. The Plain Chachalaca is sedentary breeding in the Karen Mogensen Reserve, where it is commonly observed or detected due to its typical loud call. It represents one of the most intriguing taxa of the site, since the individuals from the Karen Mogensen Reserve are part of the southernmost isolated population occurring in NW Costa Rica, and isolated from the main range of the species, which covers an area from N Nicaragua to S Texas. This population was formerly considered as White-bellied Chachalaca (*O. leucogastra*), a species living in NW Nicaragua, and more recently assigned to the present species, distinguished from the congeneric Gray-headed Chachalaca (*O. cinereiceps*) which is found elsewhere in Costa Rica. Recent studies hypothesize that this isolated population may represent an undescribed subspecies of the present species or even a distinct species, as the plumage coloration and voice differ from northern populations (del Hoyo and Kirwan 2017).

**Canivet’s Emerald** (*Chlorostilbon canivetii* [Lesson, 1832])

The presence of Canivet’s Emerald in the Karen Mogensen Reserve falls within the range documented for this species, which inhabits only the northwestern highlands of the country. Three subspecies of *C. canivetii* are known, one of which, *C. c. salvini* (Cabanis & Heine, 1860), is endemic to Costa Rica (see del Hoyo et al. 2017). Beyond the observation of this subspecies, an interesting record concerns a breeding pair (Fig. 6D) which showed traits that resembled *C. assimilis*, endemic to central and southern Costa Rica and Panama. The most relevant features, which differed from typical *C. canivetii*, were the shallower tail fork and the mostly black bill with red coloration restricted to the basis of the lower mandible. According to the information of the National Biodiversity Institute (INBio, see Calderón 2017), the populations of *C. assimilis* living in the area of the Carara National Park (some 50 km to the east of the Karen Mogensen Reserve) and southwards show similar traits to those observed by us. It is likely that the range of this peculiar population reaches also the Nicoya Peninsula. Further studies are warranted to verify the possible presence of *C. assimilis* in the Karen Mogensen Reserve.

**Yellow-naped Amazon** (*Amazona auropalliata* [Lesson, 1842])

The distribution of this Psittacid is limited to the northwestern part of the country, where it reaches the southernmost portion of its global range (del Hoyo et al. 2017b). *A. auropalliata* was resident and relatively common in the Karen Mogensen Reserve during the 1990s, but its range has contracted over years. At present it is mainly found a few kilometers away from the protected area in other localities of the Nicoya Peninsula and on the Nicoya Gulf Islands. Paradoxically, the main cause of this trend is the reforestation of the area, which had a negative impact on this species, which prefers semi-arid woodlands, savannas, and similar environments. A second, more general, cause of the progressive local decline is capture for the cage bird trade. The global population is estimated at less than 50,000 and possibly fewer than 10,000 individuals (del Hoyo et al. 2017b). For these reasons, the Yellow-naped Amazon is considered Vulnerable on a global scale (IUCN 2017).

**Three-wattled Bellbird** (*Procnias tricarunculatus* [Verreaux & Verreaux, 1853])

The Three-wattled Bellbird was encountered on different occasions from 1998 to 2016, from December to May. This species is known to be a winter visitor in the inner and eastern portion of the Nicoya Peninsula and migrates to the highlands of Central Costa Rica or to other countries for breeding (Stiles and Skutch 1989; Powell and Bjork 2004). A major threat of this species is habitat loss, especially in its wintering areas, which are poorly represented in the protected area system (Powell and Bjork 2004). Since this species is considered globally Vulnerable (IUCN 2017), mainly because of rapid population declines deriving from deforestation in its non-breeding areas (BirdLife International 2016b), our observations hopefully will stimulate further efforts to assess its presence in the Karen Mogensen Reserve, and improve conservation action within its wintering range.

**Orange-billed Nightingale-Thrush** (*Catharus aurantiirostris* Salvin, 1866; Fig. 7D)

This species is relatively easy to observe in the Karen Mogensen Reserve during the breeding season (March-August), but is more difficult to find during the rest of the year. The Costa Rican populations of Orange-billed Nightingale-Thrush are considered to belong to the subspecies *C. a. costaricensis* Hellmayr, 1902, except for those in southwestern Costa Rica, which are treated as a different subspecies *C. a. griseiceps* Salvin, 1866 (see Collar 2017). Within the Nicoya Peninsula this species is typically observed above 600 m a.s.l. (Villareal Orias et al. 2003). In the Karen Mogensen Reserve it was normally seen at around 300 m a.s.l. Apparently, the external morphology of the individuals from the surveyed area resembles that of the populations from the Central Cordilleras of Costa Rica, but the vocalizations are different. It must be noted that since the 1930s, a population was known from the highest hills of the Peninsula, isolated from those of the Central Cordilleras. This population was initially named as a distinct subspecies, *C. a. bathoica* (Bangs and Griscom 1932), but is currently merged with *C. a. costaricensis* (Collar 2017). This fact, together with the aforementioned reasons, warrant further study of morphological and molecular characters to ascertain the taxonomic status of the population inhabiting the Karen Mogensen Reserve.

**Wood Thrush** (*Hylocichla mustelina* Gmelin, 1789; Fig. 7C)

This thrush is a regular winter visitor and migrant in Costa Rica (October to April), reaching its southernmost range in Panama and NW Colombia. Thanks to the present study *H. mustelina* is reported for the first time from the Nicoya Peninsula and NW Costa Rica. The species is currently considered Near Threatened. The main threat to this species is believed to be loss and fragmentation of forests in both the breeding and wintering ranges (Collar and Sharpe 2017), along with brood-parasitism by the Brown-headed Cowbird (*Molothrus ater*; Etterson et al. 2014). Various studies have recommended that population trends be monitored to investigate the direct causes of the decline, and to increase the protection of suitable habitats in both breeding and non-breeding ranges (see BirdLife International 2017).

**Shiny Cowbird** (*Molothrus bonariensis* Gmelin, 1789)

The main range of this species is located in South America and is expanding northwards. It has recently reached Panama and, in 2004, the eastern slope of Costa Rica (Sandoval et al. 2010, Garrigues and Dean 2014). There have been one record from Cocos Island (550 km from the Pacific shore of Costa Rica; Garrigues et al. 2015) and a few records from the Southern Pacific slope of the country are known (GBIF 2017). The observation of two flocks in the Karen Mogensen Reserve in March 2016 is the first report for the Nicoya Peninsula and northwestern Costa Rica. Since Shiny Cowbird is a generalist brood parasite of many other passerine birds (approximately 30 species breeding in the Karen Mogensen Reserve are potential hosts), and has strongly negative effects on the hosts’ reproduction rates (Massoni and Reboreda 1999), further monitoring is recommended to assess whether this species has permanently colonized the area and whether conservation actions are warranted within the protected zone.

## Discussion

The species list resulting from our study is one of the most comprehensive for any site in the Nicoya Peninsula. Compared to other areas, the Karen Mogensen Reserve shows one of the highest species richness of the inland Peninsula. For instance, Villareal Orias et al. (2003) recorded 46 to 130 species from nine different zones in the Peninsula, and Solano et al. (2004) registered 158 species from three sites altogether. The relative richness of the Karen Mogensen Reserve (207 species altogether) may be attributed to its habitat heterogeneity, the presence of moist forest patches (which are otherwise rare in the region), and from its moderately complex topography, a factor known to increase bird diversity (see e.g., Mayr and Diamond 2001). Nonetheless, some National Parks of Costa Rica or the adjacent Panama show a higher overall avian species richness, typically reaching 300 to over 400 species (e.g., Slud 1980, Angehr et al. 2006). This is likely due to these areas being larger in size, but also to the general trend of lower species richness in more seasonal, drier habitats of the northern Pacific slope relative to the wetter, less seasonal southern Pacific or Caribbean forests, as pointed out by similar investigations led in Panama (Karr 1976; Ridgely and Gwynne 1989).

The present study shows that the avian community of the Karen Mogensen Reserve represents an assemblage of species typical of both the Central American dry forest environments and the moist forest of southern Pacific Costa Rica (see Stotz et al. 1996). Examples of the former group are *Morococcyx erythropygus* (Cuculidae), *Megascops cooperi* (Strigidae), *Xiphorhynchus flavigaster* (Furnariidae), *Platyrhinchus cancrominus* (Tyrannidae), *Chiroxiphia linearis* (Pipridae; Fig. 7A), and *Cyanocorax formosus* (Corvidae), while of the latter *Cyanocompsa cyanoides* (Cardinalidae) and *Euphonia hirundinacea* (Thraupidae). Similar results were reported by Solano et al. (2004) from other foothill areas of the Nicoya Peninsula. As written above, the presence of patches of moist forest and a widespread dry to moist transitional forest, together with the local occurrence of a relatively short dry season (see Sánchez-Murillo et al. 2013), distinguish this area from the greatest part of the Nicoya Peninsula, making it rather similar to the southern Pacific lowlands, which are among the richest of Costa Rica in terms of bird diversity and presence of endemic taxa.

Most (138 of 207) species were resident in the Karen Mogensen Reserve, and most of these were also breeding in the area or in the adjacent forested land (115 species plus other 14 species potentially breeding; Fig. 8A). These numbers are similar to the findings of Barrantes et al. (2016), who reported 109–135 breeding species from the dry forests of four National Parks of NW Costa Rica (Diriá, Palo Verde, Rincón de la Vieja, Santa Rosa), the smallest of which covering more than 5,000 hectares, and 104 species from the Cabo Blanco Absolute Natural Reserve (ca. 1000 hectares): the first protected area of the country. This comparison underscores the species richness of the Karen Mogensen Reserve despite its relatively small dimensions, even if it must be noted for the purpose that the surrounding and partially protected forests constitute a natural extension of the Wildlife Refuge, where some of the above mentioned species spend part of their life and may reproduce as well. The number of bird species in the Karen Mogensen Reserve increases during migration and late winter, often exceeding 160 species. From a biogeographic perspective, our study has expanded the known breeding range of two species (*G. montana* and *P. cayennensis*) formerly unknown for NW Costa Rica, and the wintering range of another, *H. mustelina*, which was never observed before within this region.

As reported above, 28 species are at the southernmost borders of their range. These populations are likely vulnerable to the future effects of climate change, and may eventually decline or even disappear, moving to more suitable areas, (see, e.g., La Sorte and Jetz 2010). An additional concern derives from several authoritative studies (see, e.g., Thomas et al. 2004, Mora et al. 2013 and related literature) reporting that exceptional climate change will occur earliest in the tropics. Since these areas have less variability in their weather than moderate-climate countries, the organisms that constitute their ecosystems are unlikely to tolerate temperatures outside their narrow range, which has existed for thousands of years, and, consequently, these taxa will reach extinction faster and earlier than those living at intermediate latitudes. As a consequence of this, most of the species recorded, starting with those that are primarily connected to tropical forests as resident breeding, should be considered at risk.

To address future conservation problems properly, it is necessary to focus on the specific ecological traits of these species. The majority of the species recorded during our study were restricted to forested environments, and this aspect underscores the importance of the Karen Mogensen Reserve as a wildlife refuge for such specialized taxa. Our analysis pointed out that 59% of the total number of species (122/207) was connected to either the understory or the middle tree level, and another 20% (42 species) is associated to middle level and canopy or open air (Fig. 8C). These proportions are similar to those in other protected areas from NW Costa Rica, as, e.g., the Bosque Nacional Diriá (Villareal Orias et al. 2003). The vertical distribution of the species is a very important factor to understand the structure of the habitat (Braker and Greene 1994), since it suggests the behaviour of the animals and their susceptibility to the modifications of vegetation. As reported by Levey and Stiles (1994), the aforementioned taxa are normally sedentary or have limited ranges, and for these reasons they are much more vulnerable to the alterations to the structure of the vegetation, which can fragment isolated populations and promote the local extinction of some species. For instance, the opening of paths, the building of infrastructures or cutting off vegetation, affect the taxa associated to these forest levels in different ways.

More generally, the species connected to the upper middle level exhibit major local horizontal or altitudinal shifting (Levey and Stiles 1994), while the birds of the understory or the understory and middle level are more vulnerable to human alterations. Since these latest taxa normally occupy restricted areas and tend to be sedentary, their local populations are more vulnerable to fragmentation, which is among the first steps toward extinction, at least on a local scale (Levey and Stiles 1994). Nonetheless, the majority of the species associated to the middle level are both ecologically and geographically restricted. For all the above mentioned reasons, most of the recorded taxa were restricted to a few protected areas, in contrast to the species typical of forest borders or not strictly related to forested environments, which tend to occupy a variety of habitats in different geographic areas (Stiles 1985). The ongoing natural forest regeneration that has characterized the surveyed area from the mid 1990s, allowed several species to colonize (e.g., *G. montana* or, likely, *C. nigrolineata*) or, mainly, recolonize the zone after many years of absence due to land use as cattle pasture. This process is likely to generate a progressive disappearance of species typical of open land, such as some Thraupidae, and also a globally threatened species (*A. auropalliata*, see above). By reducing habitat diversity, spontaneous ecological succession is likely to reduce the overall species richness of the Karen Mogensen Reserve, even if the ecological value of the whole community gradually increases.

The occurrence of three globally Vulnerable species, plus other two Near Threatened, out of the 23 listed from the all of Costa Rica, and the finding of other 44 species – mainly resident and breeding or potentially breeding in the Karen Mogensen Reserve – considered threatened on a national scale (AOCR 2005), provide a strong argument for increased conservation efforts in the area. Moreover, the occurrence and breeding of three endemic species and four endemic subspecies (see above) together with isolated populations of *Ortalis vetula* and *Catharus aurantiirostris* (Fig. 7D), disjunct from the species’ main ranges, are additional arguments for supporting the development of conservation actions in the area. The presence and breeding of large species, including *Crax rubra* (Fig. 6A) and *Penelope purpurascens* (Cracidae), provide additional evidence that the avian community of the Karen Mogensen Reserve is relatively intact, and therefore has considerable conservation value.

Furthermore, based on the forest dependence categorization, the proportion of species included in the three categories of forest dependency was roughly similar to the one detected by Barrantes and colleagues (2016) in other much broader protected areas of the Nicoya Peninsula (see above), underlining the importance of the Karen Mogensen Reserve for the local avian conservation. Most of the resident breeding species, i.e., 97 out of 129 (Fig. 8D), corresponding to almost half of the total number of recorded species (207), are restricted to forested environments. From a conservation perspective, these taxa are most affected by habitat fragmentation and more susceptible to global climatic change, since they require large mature forests to maintain stable populations (Stiles 1985). The expected increase of droughts in the region (Sheffield and Wood 2008), and the changes that could follow the increasing frequency of El-Niño Southern Oscillation (ENSO) events (Cai et al. 2014), are likely to cause longer and more intense dry seasons. The consequent intensification of the frequency of wild fires is progressively changing the structure of the northwestern Costa Rican forests (Barlow and Peres 2004) which are considered the most threatened forest ecosystem in Mesoamerica (see Janzen 1988, Huettmann 2015). These processes are altering the avian communities associated to the typical habitats of the Nicoya Peninsula (Barrantes et al. 2016).

Human induced habitat destruction and the reduction of connectivity due to the removal of isolated forest patches are likely to be additional causes of the progressive disappearance of bird populations. The genetic variability of populations limited to isolated forest patches and affected by global and local environmental alterations, may decrease in a short-term, greatly reducing their viability (see e.g. Evans and Sheldon 2008). As pointed out by Barrantes et al. (2016), the fragmentation of the originally widespread dry forest in NW Costa Rica into small isolated patches, has originated a nested pattern of bird assemblages and species, for which long-term maintenance is threatened by lack of connectivity, habitat destruction, recurrent fires, and global climatic changes. Within this problematic context, the Karen Mogensen Reserve has proven to be a reservoir of several species, many of which associated to the threatened Central American dry forests, thus playing a crucial role in the conservation of the local avifauna. The data collected during this study stimulates the development of concrete conservation actions as the creation of connections with other protected areas, the widening of the boundaries of Karen Mogensen Reserve, and the improvement of habitat restoration.

The present results underscore that further analyses are desirable, including studies aimed at clarification of (i) the taxonomic status of some local populations, (ii) the phenology of some species, (iii) the population-viability of the most vulnerable taxa, and (iv) the vulnerability of species to climate change. More generally, locally-based bird monitoring programs are essential for understanding and mitigating the effects of global change on tropical biodiversity. To measure population change and other demographic parameters – essential actions during the ongoing natural restoration of the forested environment – a long-term bird banding station should be established and supported, also considering that it would provide tools and staff for monitoring and conservation programs focused on other organisms. The presence of the recently inaugurated research station, “Italia-Costa Rica” within the Karen Mogensen Reserve represents an excellent basis for the development of this initiative.

Since birds are good environmental indicators, are relatively easy to monitor, and have a universal appeal as charismatic flagship taxa, they raise the interest of people worldwide. Therefore, short- and long-term bird monitoring and conservation initiatives need to be included in programs that involve the local communities, by promoting environmental education, capacity-building, and income generation or job creation. The educational activities should address students, villagers, conservationists, decision-makers, journalists, and other local people and should better comprise the participation of local universities, museums, and research institutions. Such integrated processes are known as some of the best examples of holistic biodiversity-monitoring programs (Latta and Faaborg 2009, Şekercioğlu 2012). The first steps in this direction have been carried out and we hope that many other will follow, in the consciousness that the spreading of scientific knowledge and the involvement of local stakeholders in the research are the main driving forces for the conservation of biodiversity.

## Conclusions

Our results show that the Karen Mogensen Reserve hosts a rich and diversified avian community, and, acting as an island in the surrounding context of NW Costa Rica, is a potential refuge for several birds, as reported in the present analysis, and for other metazoans, such as amphibians and reptiles (which will be reported in further dedicated papers), characterized by a variety of taxonomic, ecological, biogeographic, and/or conservation peculiarities. The isolation of some organisms, the existence of endemic species, the presence of taxa typical of the threatened Central American dry forest environments, and the occurrence of populations at the extreme borders of their species range, certainly encourage the development of new conservation measures together with further dedicated analyses, hopefully including a molecular investigation approach. The data collected during this study, and the characterization of the avian community of the Karen Mogensen Reserve, will be important tools for future analyses, useful to evaluate the consequences of habitat fragmentation and to monitor the effects of climate change and land use modifications on the local avifauna. We foster the creation of programs that integrate bird monitoring, ecological research, conservation initiatives, and the involvement of the local communities, by promoting environmental education, capacity-building, and income generation or job creation.
